# CB1 receptor antagonist rimonabant protects against chronic intermittent hypoxia-induced bone metabolism disorder and destruction in rats

**DOI:** 10.1007/s11325-019-02009-9

**Published:** 2020-01-02

**Authors:** Zhan-jun Dou, Xiao-Ling Gao, Yan-li Jia, Jie Chen, Jiao-Jiao Yang, Yan Chen, Shu-jie Wu, Tao Liu, Mei-ting Wang, Chong Yang, Na Zhang, Bei Wang

**Affiliations:** 1 Department of the Second Clinical Medicine, Shanxi Medical University, Taiyuan, Shanxi People’s Republic of China; 2 Department of Respiratory and Critical Care Medicine, The Second Hospital of Shanxi Medical University, No. 382, Wuyi Road, Taiyuan, 030001 Shanxi People’s Republic of China; 3 Department of Respiratory, Linfen Centrol Hospital, Linfen, Shanxi People’s Republic of China; 4 Medical Records Statistics Office, The First Hospital of Shanxi Medical University, Taiyuan, Shanxi People’s Republic of China

**Keywords:** Obstructive sleep apnea syndrome (OSAS), Cannabinoid receptor 1 (CB1R), Tartrate-resistant acid phosphatase (TRAP), Rimonabant (Ri), Chronic intermittent hypoxia (CIH)

## Abstract

**Objective:**

The endocannabinoid system (ECS) regulates bone turn-over and remodeling. Chronic intermittent hypoxia (CIH) occurring during obstructive sleep apnea (OSA) may lead to disorders of the ECS and bone metabolism abnormalities. This study aimed to investigate whether or not the cannabinoid receptor 1 (CB1R) antagonist rimonabant (Ri) alleviates bone metabolism abnormalities and bone destruction induced by chronic intermittent hypoxia (CIH).

**Methods:**

Healthy male Sprague Dawley (SD) rats (n=48) were randomly divided into 6 groups of 8 rats: 2 normal control (NC) groups, 2 intermittent hypoxia (IH) groups, and 2 IH + Ri groups. Rats in NC groups breathed room air for 4 weeks (4w NC group) and 6 weeks (6w NC group). Rats in IH groups experienced IH environment for 4 weeks (4w IH group) and 6 weeks (6w IH group). In addition to the same IH exposure, rats in IH + Ri group were given daily intraperitoneal injection of Ri at the dosage of 1.5 mg/kg/d for 4 weeks (4w IH + Ri group) and 6 weeks (6w IH + Ri group). Levels of serum tartrate-resistant acid phosphatase (TRAP, a marker of bone resorption) were determined by ELISA. Hematoxylin and eosin (HE) staining was performed on bone sections to observe the changes in bone microstructure. Expression of CB1R in bone tissue was determined by immunohistochemistry.

**Results:**

TRAP levels were higher in the 4w IH and 6w IH groups than in the 4w NC and 6w NC groups; TRAP levels were lower in the 4w IH + Ri and 6w IH + Ri groups than in the 4w IH and 6w IH groups. HE staining showed that the morphology of bone cells in the NC group was normal, but the 4w IH group had mild edema of bone cells, reduction in trabecular bone, and destruction of bone microstructure. Changes were more severe in the 6w IH group than 4w IH. The 4w IH + Ri group was slightly improved compared with the 4w IH group. The 6w IH + Ri group was improved compared with the 4w IH + Ri group. The results of immunohistochemistry showed that the expression of CB1R in IH group was significantly higher than that in NC group. The expression of CB1R in the IH + Ri group was lower than that in the IH group. With the prolongation of hypoxia, the expression of CB1R in bone cells of IH group increased. The expression level of CB1R in IH + Ri group decreased with the prolongation of intervention time. Correlation analysis showed that the expression rate of CB1R in bone cells was positively correlated with the level of TRAP in serum.

**Conclusion:**

CIH increases serum TRAP levels and triggers metabolic bone disorder by activating bone CB1R. Intervention with CB1R antagonist (rimonabant) reduces the bone dysmetabolism in the CIH rat model.

## Introduction

Obstructive sleep apnea syndrome (OSAS) causes chronic intermittent hypoxia (CIH) along with a series of pathological conditions such as sympathetic system activation, oxidative stress, and a cascade of systemic inflammation [1]. The hypoxia-reoxygenation pattern seen with CIH is a main pathophysiological feature of OSAS [2] and may result in damage to a number of target organs [3]. Over time, OSAS is associated with numerous comorbid conditions including hypertension, hyperlipidemia, insulin resistance, abnormal glucose tolerance, obesity, asthma, myocardial infarction, heart failure, and stroke. Data from numerous countries have shown a very close correlation of OSAS with increasing age and double the prevalence of the disease in men compared to women [4–6]. A number of studies, including a systematic review of 24 studies published in 2017, estimate the overall prevalence of obstructive sleep apnea to be between 9% and 38% in the general adult population [7–9]. 

Accumulating evidence has shown that a hypoxic environment may cause abnormal changes in bone metabolism resulting in osteoporosis [10], a systemic metabolic bone disease characterized by inferior quality of bone and damage to bone microstructure with increased risk for bone fragility and fractures. Patients with OSAS may represent a group at risk for bone resorption and loss of bone mineral density. It is important to evaluate patients with OSAS for possible bone loss [11]. 

The endocannabinoid system (ECS) is an endogenous signaling system involved in the regulation of various physiological functions in vivo [12]. The ECS is composed of the cannabinoid receptors CB1R and CB2R and their degradation enzyme and synthetase. These two receptors are G protein-coupled receptors [13]. CB1R plays an important role in adjusting bone mass and bone remodeling. [14–16]. A previous clinical study by this research group showed that patients with OSAS may develop a disorder of the ECS, which may be relieved by continuous positive airway pressure (CPAP) treatment. OSAS appears to cause an increase in activation of CB1R in ECS. However, whether or not this increase receptor activation may result in a metabolic disorder of bone is unknown. Furthermore, it is not known whether or not rimonabant, an efficient selective CB1R antagonist, can relieve the bone dysmetabolism seen in patients with OSAS.

Metabolic disorders of bone are caused by an increase in osteoclasia and a decrease in osteogenesis such that bone resorption is stronger than bone formation. Serum tartrate-resistant acid phosphatase (TRAP) is widely accepted in clinical practice and scientific research as a highly specific and sensitive index of bone metabolism reflecting changes in bone resorption [17]. Research data have indicated that the CIH of OSAS may affect the kidney, gastrointestinal tract, osteoblasts, osteoclasts, sympathetic nerves, leptin, and an inflammatory response resulting in abnormal bone metabolism [18, 19]. However, changes in the biochemical indexes of bone metabolism in an environment of CIH are not fully understood.

This research therefore aimed to establish a rat model of CIH in order to study the mechanism of action of the ECS in bone metabolism. This rat model then provides the opportunity to inject intraperitoneal rimonabant while measuring bone metabolism by serum TRAP levels along with examination of cell morphology in bone tissue, changes in bone microstructure, and expression of CB1R in osteocytes. 

## Materials and method

### Animal care

Forty-eight male healthy SD rats (weighing from 450 to 500 g) aged from 8 to 10 weeks were purchased from the animal center of Shanxi Medical University. Animals were maintained in clean cages with controlled temperature at 23 ± 2 °C in 12/12 h light–dark cycles (lights on 8:00 am). They were provided free access to food and water ad libitum. All rats were weighed daily for 3 days before the experiment.

## CIH model

About 48 male healthy SD rats were randomly divided into 6 groups with 8 rats in each: 2 normal control (NC) groups, 2 intermittent hypoxia (IH) groups, and 2 IH + Ri groups. Rats in NC groups were kept breathing room air for 4 weeks (4w NC group) and 6 weeks (6w NC group). Rats in IH groups experienced IH environment for 4 weeks (4w IH group) and 6 weeks (6w IH group). Apart from exposure to the same IH as in IH groups, rats in IH + Ri group were given daily intraperitoneal injection of Ri at the dosage of 1.5 mg/kg/d for 4 weeks (4w IH + Ri group) and 6 weeks (6w IH + Ri group). The IH group rats were exposed to sham or CIH exposure. Rats were housed as normal in standard cages placed within commercially designed environmental chambers for daily gas treatments. A gas control system was used to regulate the flow of oxygen and nitrogen into the chamber. Ambient oxygen was servo-controlled to generate intermittent hypoxia. During a 2-min circle, nitrogen was first filled into the chamber at a set rate to reach a fraction of inspired oxygen (FiO2) at 8% from 21% within 30 s. Then, compressed air was introduced into the chamber at a rate of 10 L/min to achieve an FiO2 of 21% within 50 s, and compressed air at 5 L/min was filled after that to maintain the level of 21% oxygen for 40 s until a new cycle. Rats were placed into the chamber for 30 cycles per hour, 8 h per day for 4 or 6 consecutive weeks. Rats in the CG were kept in the chamber with FiO2 of 21% all throughout the experiment [20]. The oxygen concentration in the chambers was tested by a portable oxygen analyzer. This study was carried out in strict accordance with the recommendations in the Guide for the Care and Use of Laboratory Animals of the National Institutes of Health. The animal use protocol has been reviewed and approved by the institutional animal care and use committee of The Second Hospital of Shanxi Medical University.

Rats in the IH group received a daily intraperitoneal injection of the CB1 receptor antagonist, rimonabant (1.5 mg/kg/d), a day before they were placed into the chronic intermittent low-oxygen chamber and for 4 or 6 weeks in total. The NC groups were exposed to only compressed air. An electrode was inserted into the cabin ventilation to confirm all changes in oxygen content. Oxygen content in the hypoxia cabin fluctuated between 8% and 21% adjusted by the gas flow-rate.

## Enzyme-linked immunosorbent assay (ELISA)

After 4 or 6 weeks, the rats were anesthetized by 10% chloral hydrate, 0.3 ml/100 g, abdominal anesthesia. Extract serum, determination of TRAP in serum by enzyme-linked immunosorbent assay. The six test tube concentrations were diluted to 160 pg/ml, 80 pg/ml, 40 pg/ml, 20 pg/ml, 10 pg/ml, and 0 pg/ml. Add 50 ul of standard samples of different concentrations to the standard wells and set blank holes and holes to be tested. Add 40 ul of sample to the hole to be tested. Biotinylated anti-TRAP antibody was added, and blank wells were not loaded. The sampled micropores were sealed with a sealing film and placed in a 37 °C incubator for 30 min. The washing solution was diluted 20 times and then used. Remove the sealing film, discard the liquid, add the washing solution, let stand for several tens of seconds, discard, and dry. All the wells except the blank wells were incubated with the enzyme labeling reagent and washed again. Color development, first add the color developer A50ul to each well and then add the color developer B50ul, gently shake and mix, and develop color at 37 °C for 15 min. Termination: Blue turns yellow and the reaction is terminated, and absorbance (OD value) is measured within 15 min.

## Hematoxylin and eosin staining (HE)

After 4 or 6 weeks, the rats were anesthetized. After removing the rat tibia, fix it with 4% paraformaldehyde for 24 h, use 4 W decalcifying solution, change the decalcifying solution once every 2 days until the bone tissue becomes soft, and then cut the material from the coronal plane. The alcohol gradient was dehydrated, and after the xylene was transparent, it was embedded under a paraffin machine having a melting point of 60–62 °C, and finally the continuous section was about 4–5 μm thick. Morphological changes in bone tissue were observed by HE staining. The dried slices were soaked in xylene, dewaxed for 10 min for three times, and then put into 100% and 95% alcohol, respectively, for 2 min with two times each. After washing with water, slices were rinsed by hematoxylin for 5 min and washed for three times, followed by a 1% hydrochloric acid alcohol differentiation for 1 to 2 s, 0.5% eosin coloring for 10 s to 1 min, 70%,85% ethanol both for 2 min, and 95% ethanol for 3 to 5 s. After that, slices were dried over anhydrous ethanol for 1 min followed by gradient alcohol dehydration and the final xylene for both 2 min with three times each. Then slices were took out and wiped, and a drop of mounted neutral gum was added. Finally, optical microscope was used to observe the change of bone tissue morphology and structure at 100 magnifications.

## Immunohistochemical analysis

The expression of CB1 receptor in the bone cells was detected by immunohistochemistry. The slices were dehydrated in different concentrations of ethanol for 5 min each and then put into 3% peroxide solution for 10 min, distilled water for three times with 5 min each and phosphate buffer saline (PBS) liquid for 3 times with 2 min each. The 5% albumin from bovine serum albumin (BSA) liquid is added to make the antigen closed, and the incubator is incubated for about 20 min. CB1 antibody (concentration of 1:50) is evenly added, covering the bone tissue with antibody contact, overnight at 4 °C. After PBS rinsing, the second antibody placed in 37 °C incubator for 30 min is added. Then PBS was washed three times with 2 min each. Streptavidin-biotin complex (SABC) was joined into the wet box incubation for 20 min, and then PBS was washed for four times.

Diaminobenzidine (DAB) was used to color for 5 min, to dye the extent with distilled water rinse termination. Hematoxylin staining solution was added for 15 s, rinsed with water for 5 min, and then put in the differentiation in hydrochloric acid alcohol. Gradient alcohol dehydration, transparent, orderly mounting was conducted under the microscope. The result showed that the negative control was only two anti-negative controls. ImagePlus4.0 image analysis software was used to carry out semiquantitative analysis of and CB1 receptor in the bone tissue according to the staining area and intensity.

## Real-time fluorescence quantitative PCR

Real-time fluorescence quantitative polymerase chain reaction (PCR) was also used to detect the expressions of CB1 and TRAP in bone tissues of rats. Primers were designed using Primer 3.0 software (http://frodo.wi.mit.edu/primer3). Total RNA was extracted from brain tissues of rats using Trizol reagent (Invitrogen, USA) following the manufacturers instruction. Relative mRNA expression levels were determined by SYBR Green I kit (Biotechs, China). The amplification procedure was 2 min at 50 °C and 10 min at 94 °C followed by 40 cycles of 94 °C for 40 s, 60 °C for 30 s, and 72 °C for 30 s. Data were reported as values normalized by the housekeeping gene b-actin. Genetic relative quantitative was calculated with 2^-ΔΔCt^ methods.

## Result

### TRAP levels in serum and bone tissues were increased by the CIH, which was attenuated by rimonabant treatment

**Fig. 1:**
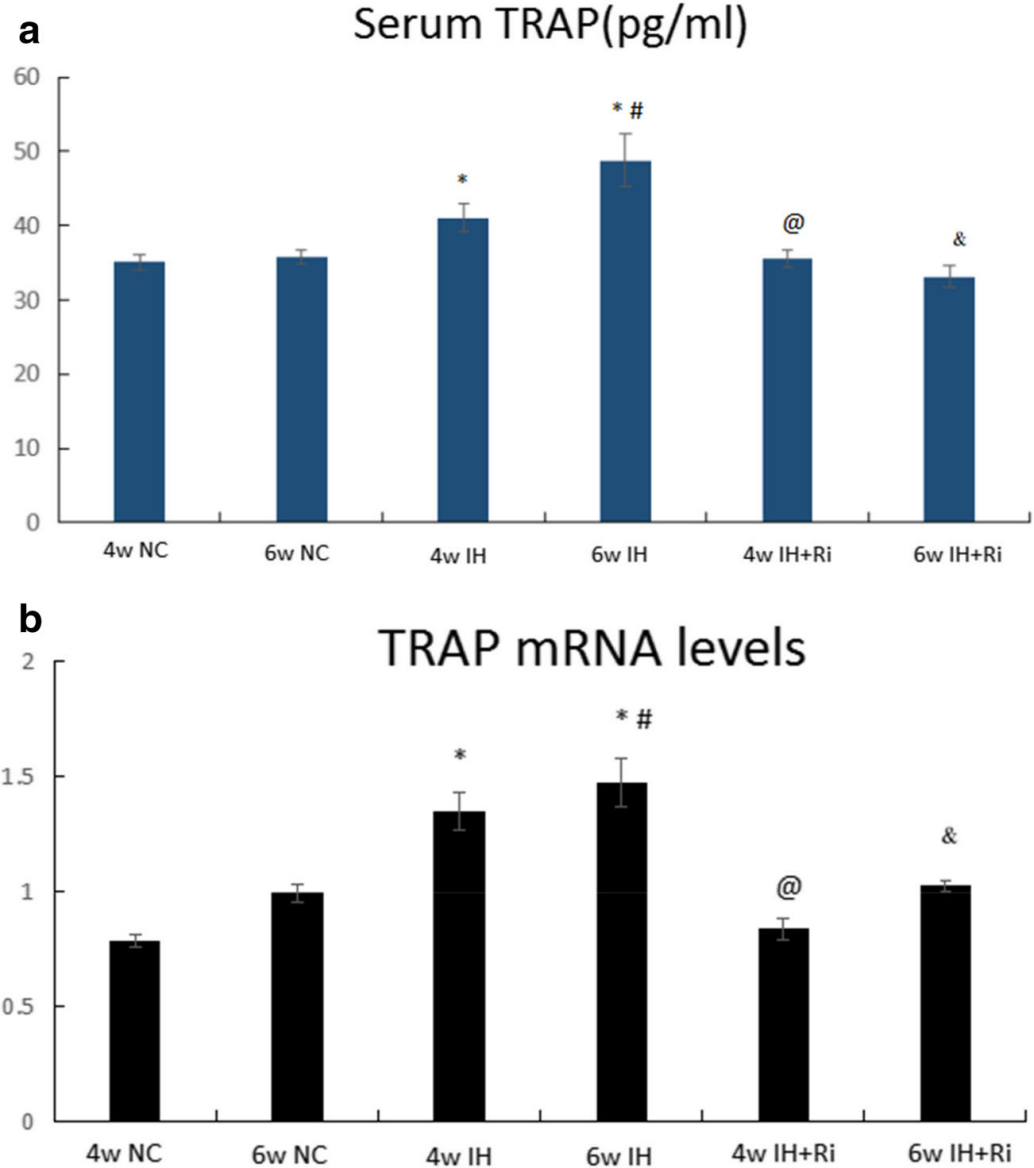
Determination of TRAP in serum (**a**) and bone tissues (**b**) of rats in each group by double antibody sandwich method.*IH group compared with NC group, *P* < 0.001; #6w IH group compared with 4w IH group, *P* < 0.001; @ 4w IH group compared with 4w IH + Ri group, *P* < 0.001; & 6w IH group compared with 6w IH + Ri group, *P* < 0.001

Although hypoxic exposure causes abnormal changes in bone metabolism, it is unclear whether CIH leads to bone disorder. To answer this question, we determined the levels of TRAP enzyme by ELISA in serum and bone tissues of rats exposed to CIH. We found that CIH exposure for 4 and 6 weeks increased the levels of TRAP in both serum and bone tissues. The levels of TRAP were further increased by 6 weeks of CIH exposure compared to 4 weeks of CIH exposure. Treatment with rimonabant significantly reduced CIH-induced increase in TRAP in both serum and bone tissues. Moreover, rimonabant treatment for 6 weeks was efficient to reduce the TRAP compared to 4 weeks of rimonabant treatment in response to CIH exposure. These results demonstrate that CIH exposure increases the levels of TRAP in both serum and bone tissues and these effects are reduced by CB1 antagonist rimonabant. (Fig. 1).

### CIH increases bone destruction in bone tissue, which is attenuated by rimonabant treatment

**Fig. 2:**
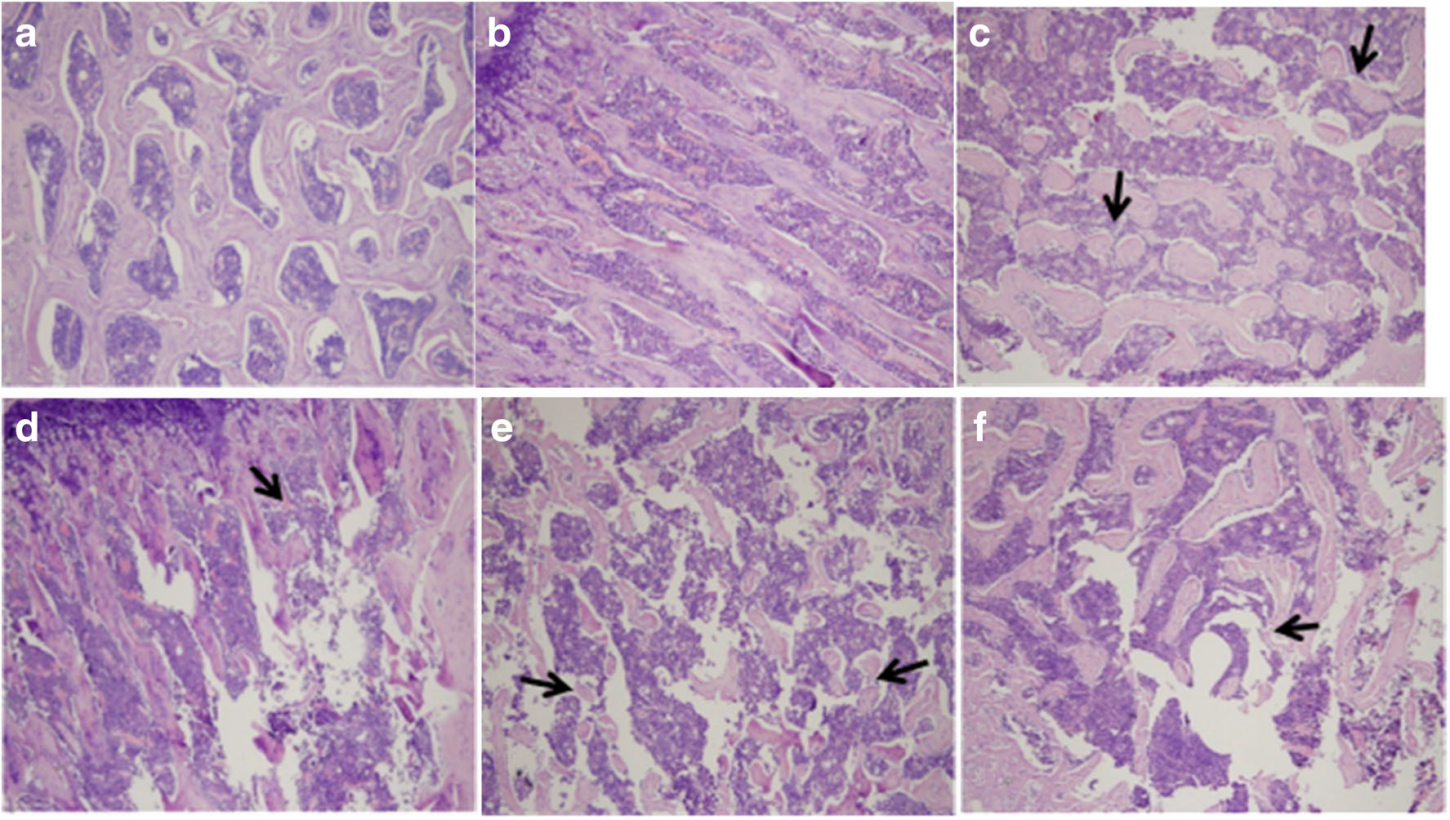
Morphological changes of bone tissue in each group were observed by HE staining (HE **×** 100). (**a**) 4w NC group. (**b**) 6w NC group. The bone tissue in 4w and 6w NC group is arranged in neat columns, and the bone trabecula structure is intact. (**c**) 4w IH group. A small amount of bone tissue is lost, their arrangement is disordered, the number is decreased, and the cells are edema. (**d**) 4w IH + Ri group. The trabecula structure is better than before. (**e**) 6w IH group. The bone tissue loss is more serious, trabecular structure is obviously damaged, the number is significantly reduced, and bone cells are highly edema. (**f**) 6w IH + Ri group. The destruction of bone tissue is significantly reduced

We observed the morphology of rat bone by HE staining. We found that CIH exposure increased bone destruction at 4 and 6 weeks of exposure. The 6-week damage of CIH exposure was more severe than the 4-week CIH exposure. Treatment with rimonabant significantly reduced CIH-induced bone destruction. In addition, rimonabant treatment for 6 weeks was effective in reducing bone damage compared to the 4-week rimonabant treatment group. These results indicate that CIH exposure increases bone tissue destruction and that the CB1 antagonist rimonabant reduces these effects (Fig. 2,bone destruction as indicated by the arrow).

### CIH increases CB1R levels in bone tissue, which is attenuated by rimonabant treatment

**Fig. 3:**
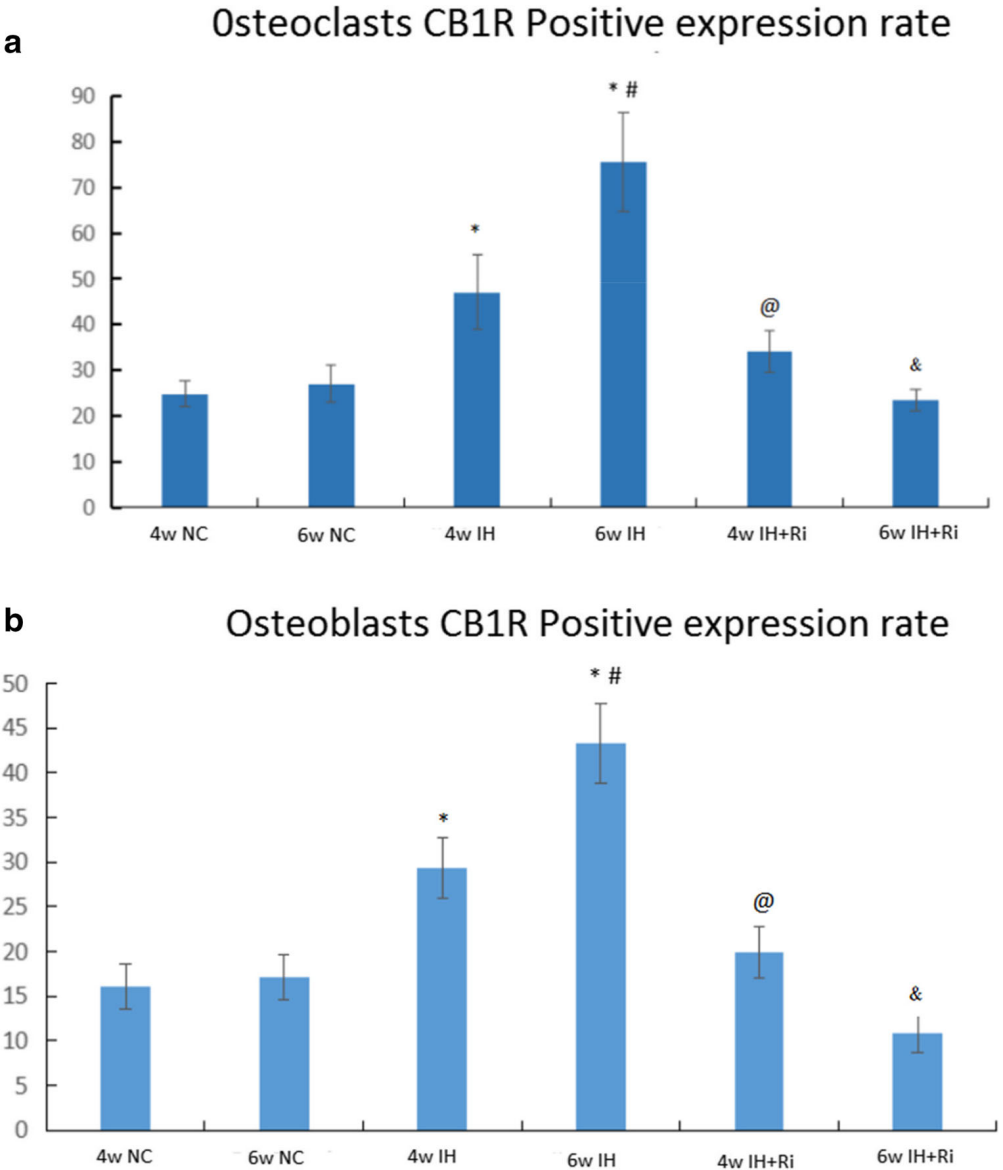
Positive expression rate of CB1 receptor in osteoclasts (**a**) and osteoblasts (**b**) of rats in each group. *IH group compared with NC group, *P* < 0.001; #6w IH group compared with 4w IH group, *P* < 0.001; @ 4w IH group compared with 4w IH + Ri group, *P* < 0.001;& 6w IH group compared with 6w IH + Ri group, *P* < 0.001

**Fig. 4:**
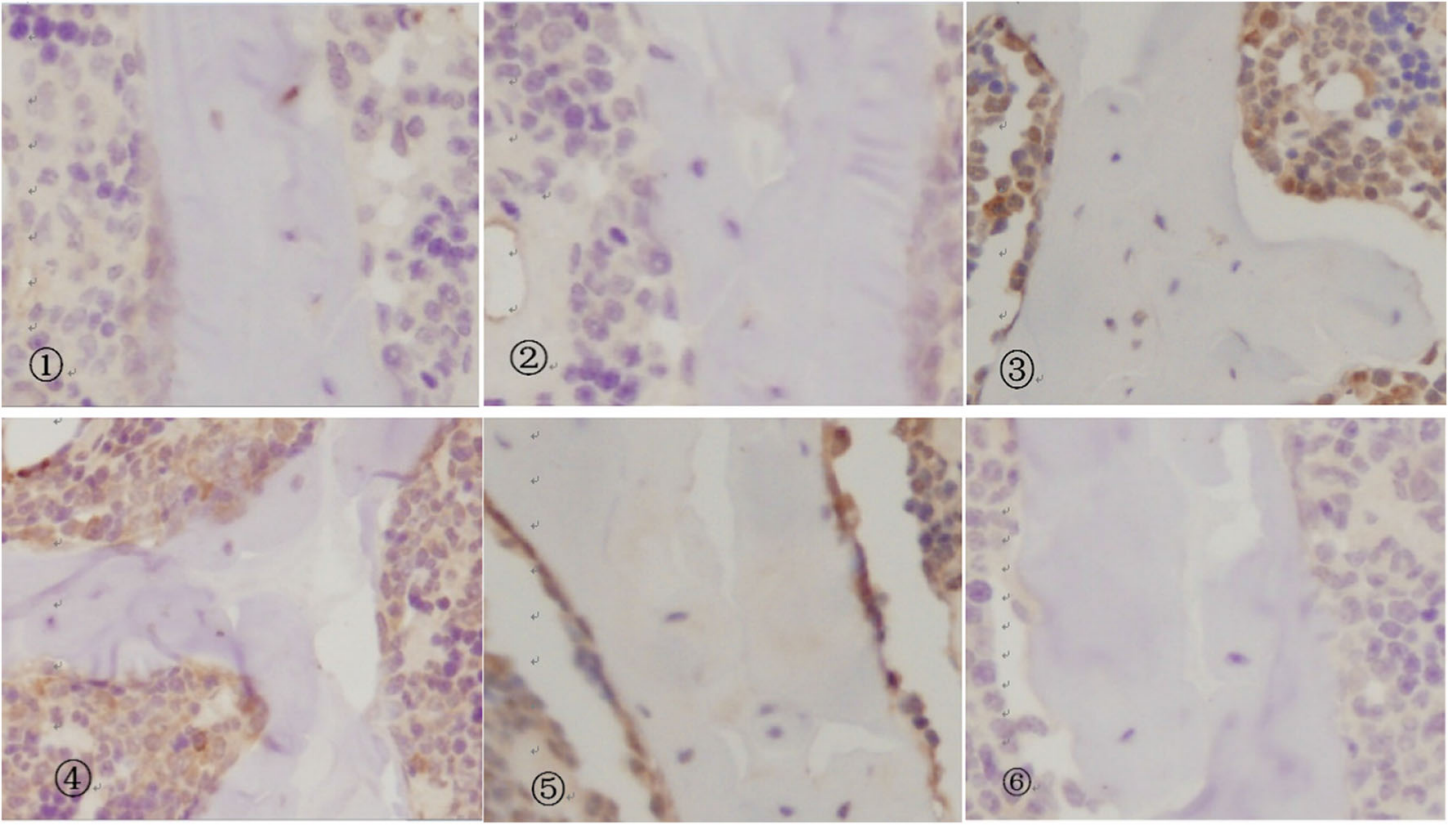
Immunohistochemistry results of bone tissue in each group (immunohistochemistry, × 400). ①, 4w NC group; ②, 6w NC group; ③, 4w IH group; ④, 4w IH + Ri group; ⑤, 6w IH group; ⑥, 6w IH + Ri group

**Fig. 5:**
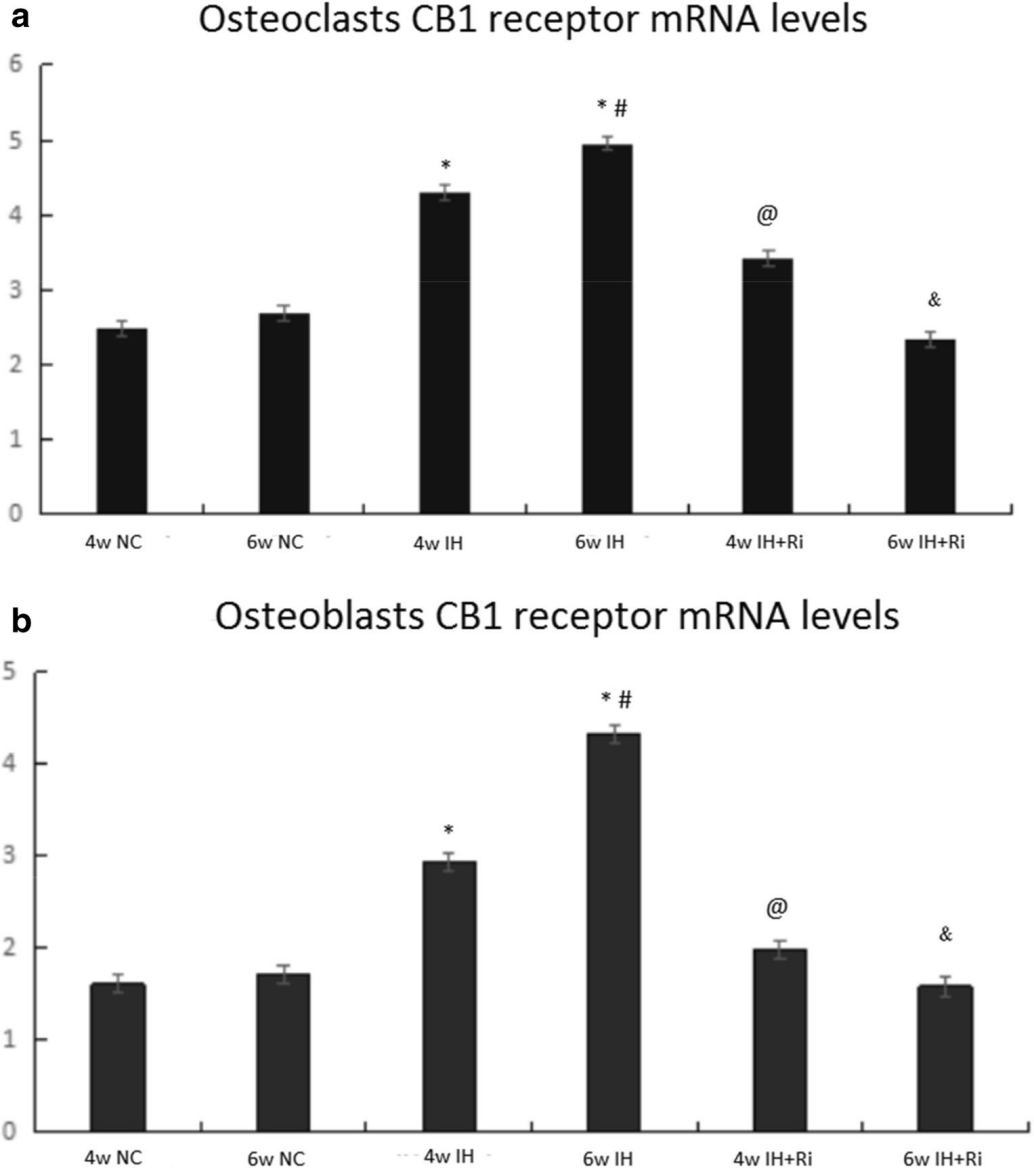
Positive expression rate of CB1 receptor in osteoclasts (**a**) and osteoblasts (**b**) of rats in each group.*IH group compared with NC group, *P* < 0.001; #6w IH group compared with 4w IH group, *P* < 0.001; @ 4w IH group compared with 4w IH + Ri group, *P* < 0.001;& 6w IH group compared with 6w IH + Ri group, *P* < 0.001

We determined the levels of CB1R by immunohistochemistry in bone tissues of rats exposed to CIH. We found that CIH exposure for 4 and 6 weeks increased the levels of CB1R in both serum and bone tissues. The levels of CB1R were further increased by 6 weeks of CIH exposure compared to 4 weeks of CIH exposure. Treatment with rimonabant significantly reduced CIH-induced increase in CB1R in both serum and bone tissues. Moreover, rimonabant treatment for 6 weeks was efficient to reduce the CB1R compared to 4 weeks of rimonabant treatment in response to CIH exposure. These results demonstrate that CIH exposure increases the levels of CB1R in both serum and bone tissues and these effects are reduced by CB1 antagonist rimonabant (Figs. 3, 4, and 5).

### Correlation comparison

Pearson correlation analysis showed that there was a positive correlation between ECs receptor CB1R expression level and serum TRAP in OC and OB (OC/r value 0.836, *P* < 0.001)/(OB/r value 0.806, *P* < 0.001) (Table 1).

**Table 1:** Correlation between CB1R expression rate and serum TRAP levels

Index	r	P
TRAP(OC)	0.836	*P* < 0.001
TRAP(OB)	0.806	*P* < 0.001

Note: *OB* osteoblasts, *OC* osteoclasts

## Discussion

In summary, the OSAS pathophysiologic process mainly involves abnormal negative pressure fluctuation in the chest, sleep structure disorder, and repeated exchange of hypoxia and reoxygenation. The most significant is CIH repeatedly occurring at night. All the above factors jointly play their roles and result in hyperfunction of sympathetic nerves, enhancement of systemic inflammation, and increase of oxidative stress and thus further induce the synthesis and release of a variety of inflammatory factors and active medium. Finally all the target organ systems are damaged. The epidemiological survey indicates that [21] habitual snoring disease (the common syndrome of sleep related breathing disorder) is the risk factor of fracture healing delay and OP development.

In this test, CIH rat model is adopted to simulate OSAS pathophysiologic process. It could basically satisfy requirements of this test. Result acquired after observing HE staining demonstrates that it can be observed using a light microscope that for rats in CIH environment, their bone structures develop abnormal changes, which are displayed through disordered bone trabecula structure arrangement and decrease in quantity. In addition, the degree of abnormal changes in bone structure is related to extension of time of CIH. After using CB1R antagonist-rimonabant for intervention, abnormality of bone structures is relieved, which is displayed through that quantity and structure of bone trabecula have certain reserve and osteocytes only have slight swelling. Therefore, we get that such special pathological changes of OSAS in CIH may cause pathological changes in bone tissue.

That OSAS patients develop ECS disorder have been found in the early clinical study of this research group [22, 23], which is displayed through CB1R overexpression, which results in many enzymes used for synthesis of active substances and further results in increase in action of ECS. Considering that for ECS, CB1R has the receptor damaging functions, which detect the expression of CB1R to reflect changes in ECS system function. In this study, the immunohistochemical method is adopted to detect expression of CB1R in CIH rat bone tissue. The image analysis result demonstrates that CB1R does scatter in bone tissue. Compared with normal tissue, level of expression of CB1R in IH group increases and that of 6w IH is higher than that in 4w IH. However, in IH group where rimonabant is used for intervention, expression of CB1R decreases, and the decrease in 6w IH group is more obvious. There are no obvious changes among NC groups. Thus, we could know that CIH could increase level of expression of CB1R in bone tissue and the level of expression is related to duration of hypoxia and intervention.

Considering that specific CB1R antagonist could block combination between CB1R and its ligand, and in this test, the serum TRAP level of the bone absorption indicator has been detected. Carrying out the hypoxia intervention using 1.5 mg/kg/d rimonabant before modeling, the result indicated that after intervention, expression of CB1R was relieved and level of the serum TRAP declined. In addition, bone tissue damages were also relieved. It means rimonabant plays a role in relieving ECS disorder and bone metabolic disorder caused by CIH through blocking CB1 receptor. Meanwhile, we also carried out the correlation analysis between the level of expression of CB1R and the level of the serum TRAP, and then we found the obvious correlation. CIH could cause the increase in expression ratio of CB1 in osteocytes and level of the serum TRAP, as well as certain damages to bone tissue structure. Moreover, close correlation exists between them.

It is demonstrated from both sides that CIH caused by OSAS could cause ECS disorder and finally result in bone metabolic disorder and abnormal bone structure. Studies [24] have shown that both CB1 and CB2 receptors are expressed on mouse osteoblasts and bone marrow mesenchymal stem cells. Studies [25] have also proved that decrease in expression of CB1 receptor could relieve bone metabolic abnormality caused by ECS disorder, which is the same as the result of this test.

In short, no matter for either osteoclast or osteoblasts, CIH could cause the increase in expression of CB1R and further result in increase in the level of TRAP activation, which proves that CIH does cause ECS disorder and cause bone metabolic disorder through activating CB1 receptor in bone tissue. After using CB1 receptor antagonist for intervention, expression of CB1 receptor is weakened, and the level that represents the bone absorption metabolic indicator also decreases. Meanwhile, damages to bone trabecula are relieved. The above indicates that the rimonabant plays a certain role in changes in bone mass and bone structure caused by CIH and lowers down risks of CIH rat model developing ECS disorder and bone metabolic abnormality and hints that effective suppression of the expression level of CB1R could provide a new way for prevention and treatment of OSAS and OP. However, we did not study changes in CB1 receptor and bone tissue under different hypoxia concentration, which shall be further discussed in the future study.
